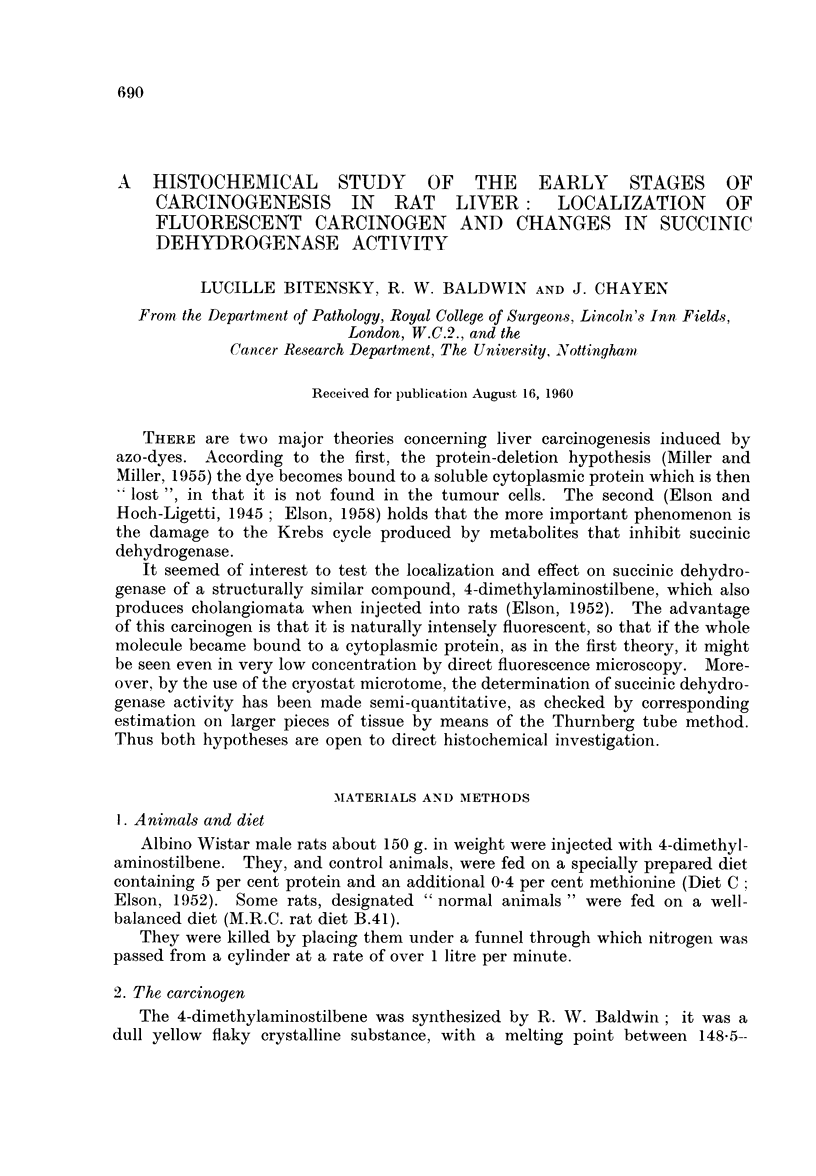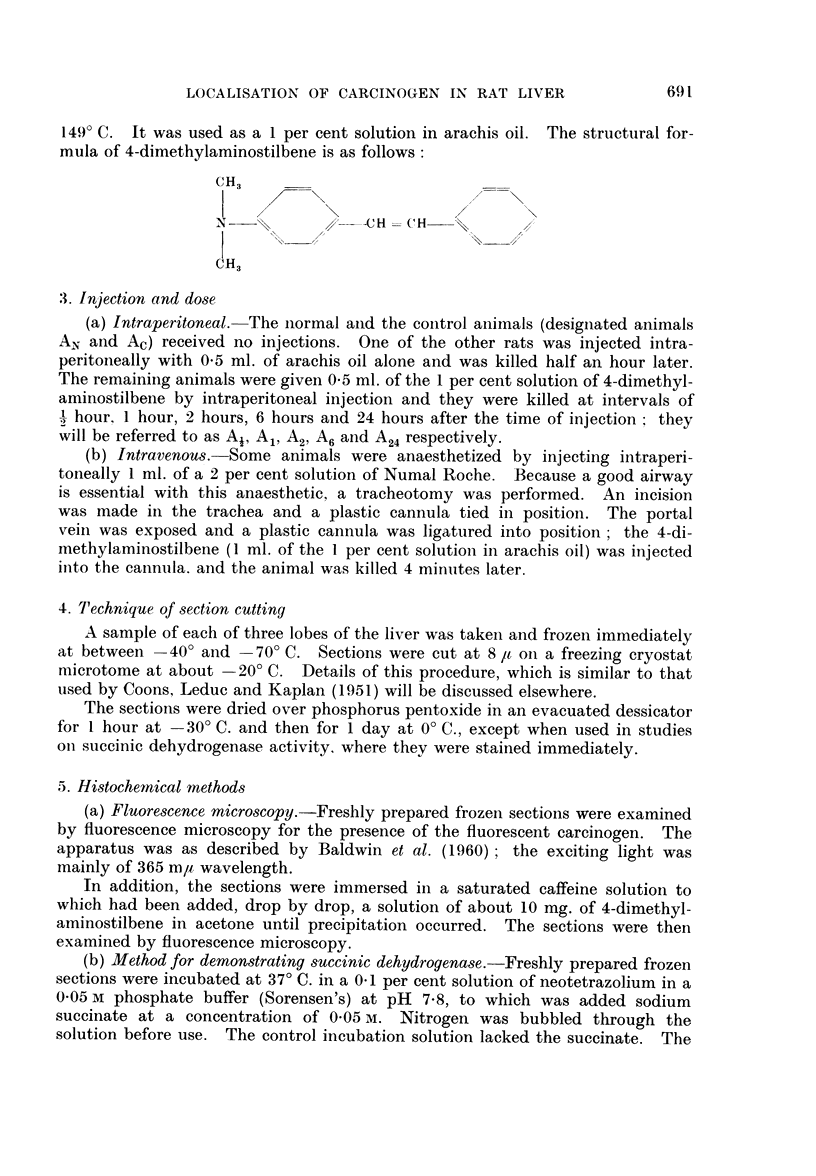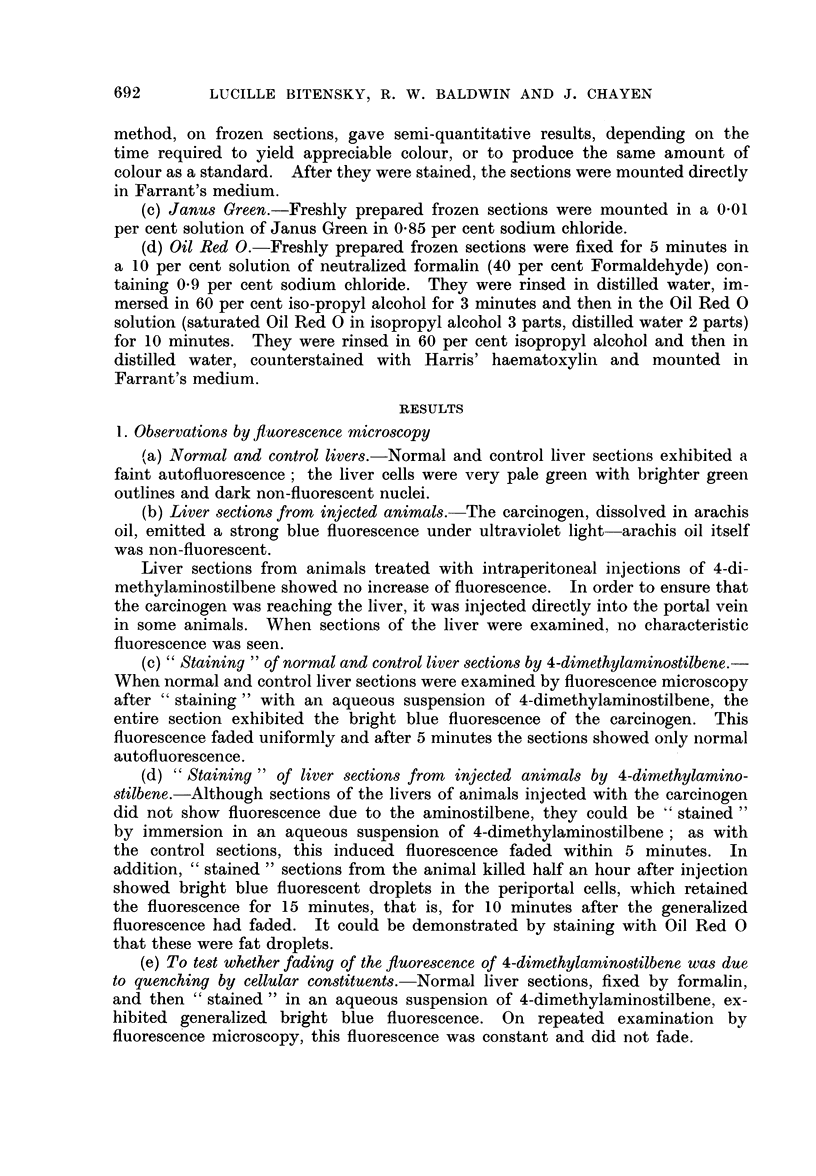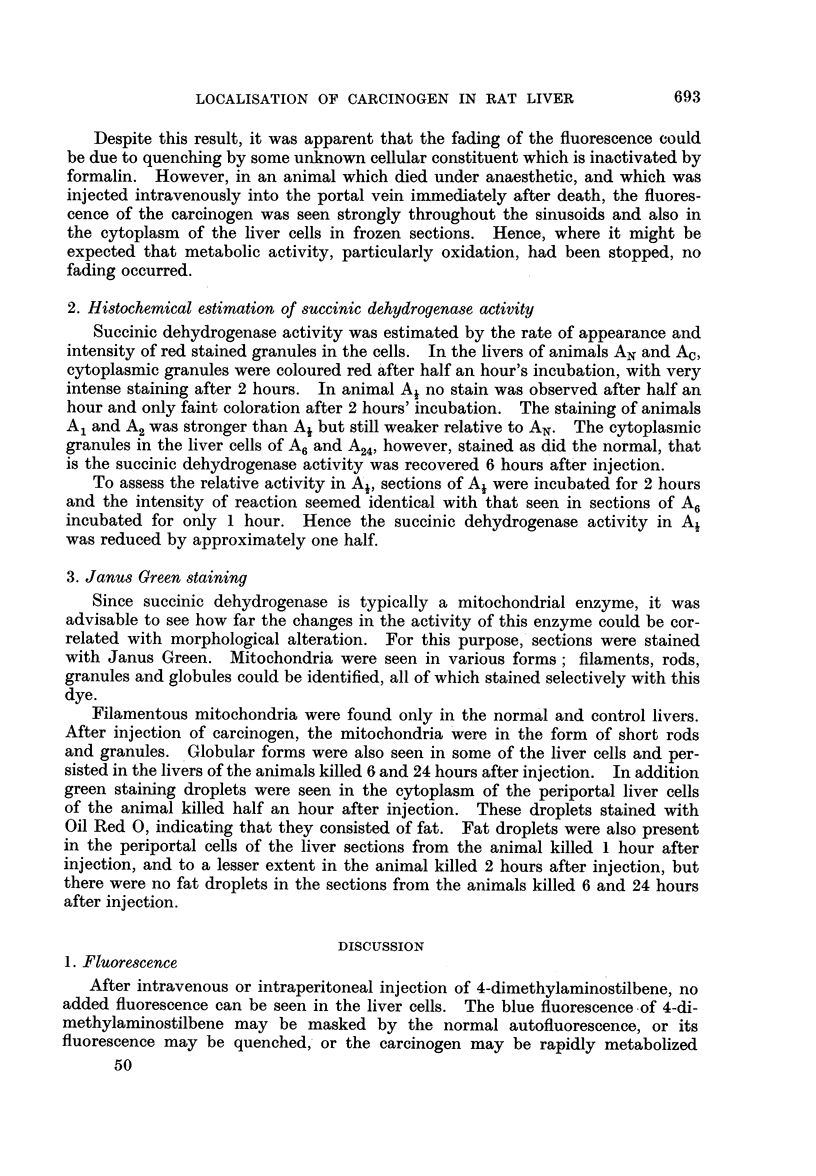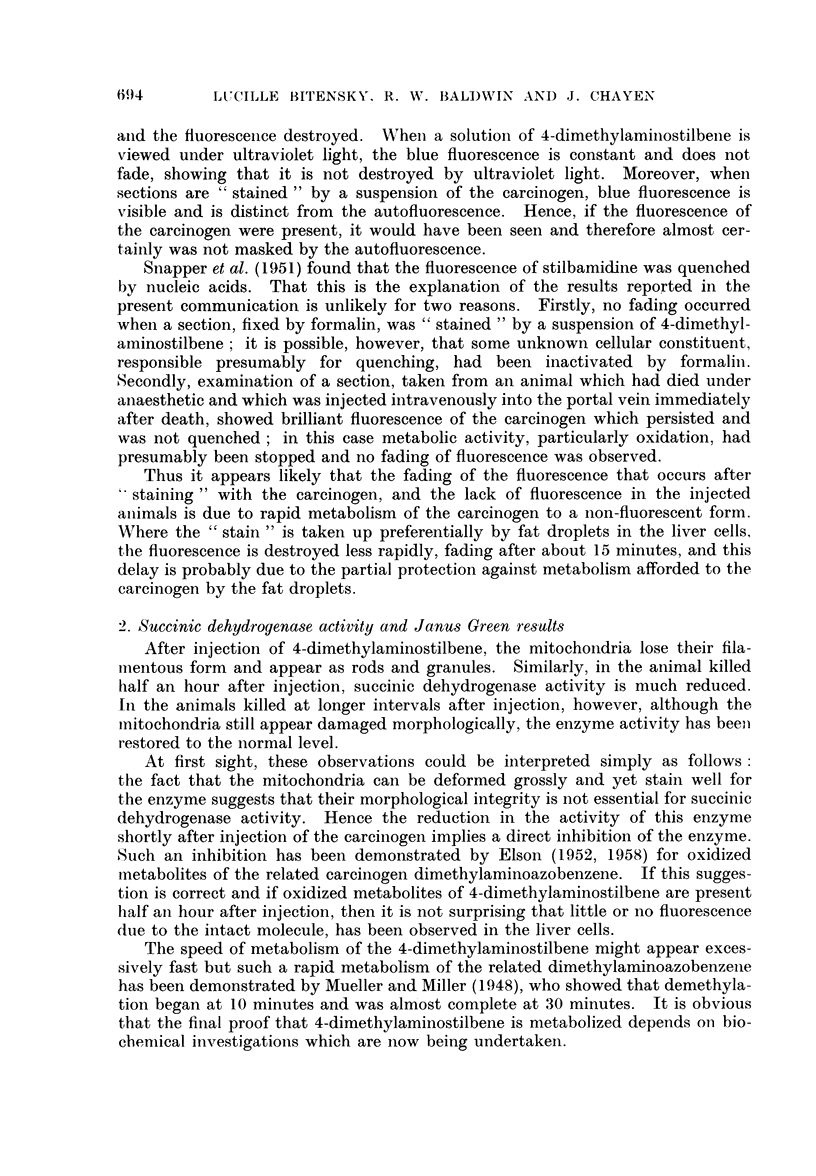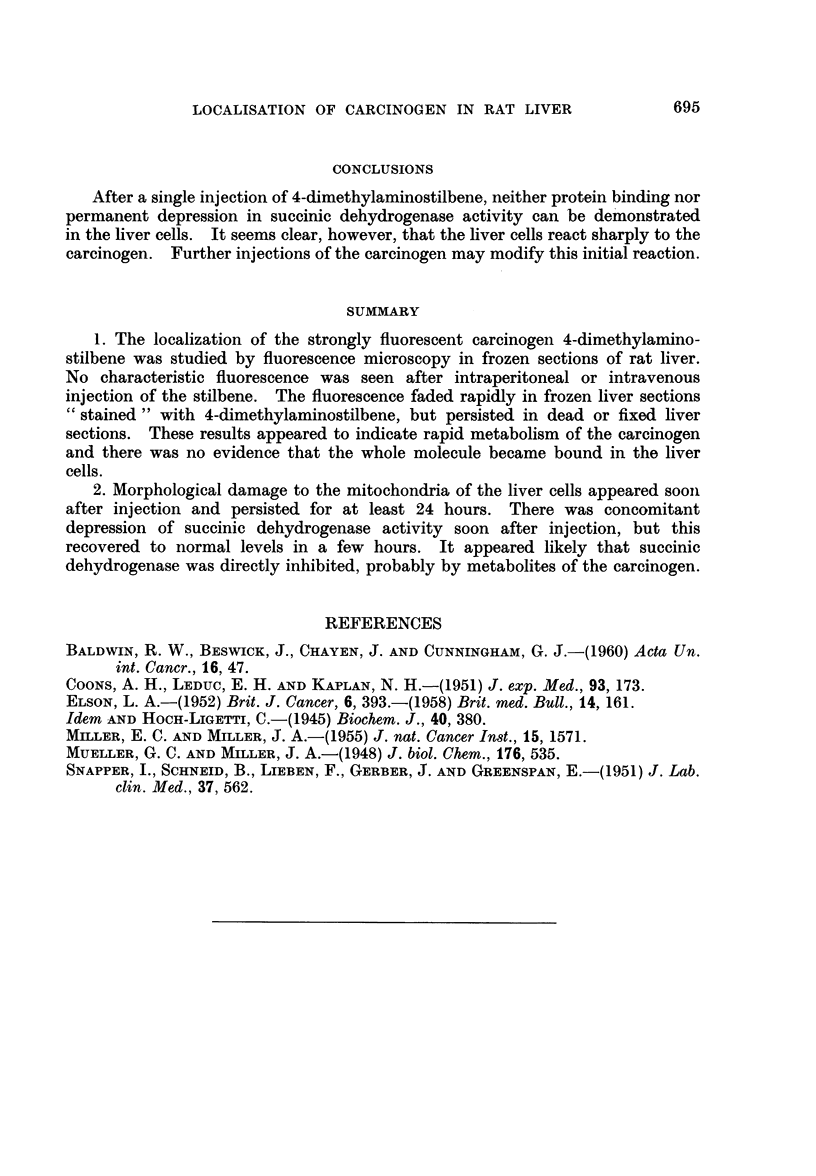# A Histochemical Study of the Early Stages of Carcinogenesis in Rat Liver: Localization of Fluorescent Carcinogen and Changes in Succinic Dehydrogenase Activity

**DOI:** 10.1038/bjc.1960.77

**Published:** 1960-12

**Authors:** Lucille Bitensky, R. W. Baldwin, J. Chayen


### A HISTOCHEMICAL STUDY OF THE EARLY STAGES OF CARCINOGENESIS IN RAT LIVER: LOCALIZATION OF FLUORESCENT CARCINOGEN AND CHANGES IN SUCCINIC DEHYDROGENASE ACTIVITY

LUCILLE BITENSKY, R. W. BALDWIN AND J. CHAYEN

*From the Department of Pathology, Royal College of Surgeons, Lincoln's Inn Fields, London, W.C.2., and the Cancer Research Department, The University, Nottingham*

Received for publication August 16, 1960

There are two major theories concerning liver carcinogenesis induced by azo-dyes. According to the first, the protein-deletion hypothesis (Miller and Miller, 1955) the dye becomes bound to a soluble cytoplasmic protein which is then "lost", in that it is not found in the tumour cells. The second (Elson and Hoch-Ligetti, 1945; Elson, 1958) holds that the more important phenomenon is the damage to the Krebs cycle produced by metabolites that inhibit succinic dehydrogenase.

It seemed of interest to test the localization and effect on succinic dehydrogenase of a structurally similar compound, 4-dimethylaminostilbene, which also produces cholangiomata when injected into rats (Elson, 1952). The advantage of this carcinogen is that it is naturally intensely fluorescent, so that if the whole molecule became bound to a cytoplasmic protein, as in the first theory, it might be seen even in very low concentration by direct fluorescence microscopy. Moreover, by the use of the cryostat microtome, the determination of succinic dehydrogenase activity has been made semi-quantitative, as checked by corresponding estimation on larger pieces of tissue by means of the Thurnberg tube method. Thus both hypotheses are open to direct histochemical investigation.

### MATERIALS AND METHODS

### 1. *Animals and diet*

Albino Wistar male rats about 150 g. in weight were injected with 4-dimethylaminostilbene. They, and control animals, were fed on a specially prepared diet containing 5 per cent protein and an additional 0·4 per cent methionine (Diet C; Elson, 1952). Some rats, designated "normal animals" were fed on a well-balanced diet (M.R.C. rat diet B.41).

They were killed by placing them under a funnel through which nitrogen was passed from a cylinder at a rate of over 1 litre per minute.

### 2. *The carcinogen*

The 4-dimethylaminostilbene was synthesized by R. W. Baldwin; it was a dull yellow flaky crystalline substance, with a melting point between 148·5–

149° C. It was used as a 1 per cent solution in arachis oil. The structural formula of 4-dimethylaminostilbene is as follows :

3. *Injection and dose*

(a) *Intraperitoneal.*—The normal and the control animals (designated animals $A_N$ and $A_C$) received no injections. One of the other rats was injected intraperitoneally with 0·5 ml. of arachis oil alone and was killed half an hour later. The remaining animals were given 0·5 ml. of the 1 per cent solution of 4-dimethylaminostilbene by intraperitoneal injection and they were killed at intervals of ½ hour, 1 hour, 2 hours, 6 hours and 24 hours after the time of injection ; they will be referred to as $A_{\frac{1}{2}}$, $A_1$, $A_2$, $A_6$ and $A_{24}$ respectively.

(b) *Intravenous.*—Some animals were anaesthetized by injecting intraperitoneally 1 ml. of a 2 per cent solution of Numal Roche. Because a good airway is essential with this anaesthetic, a tracheotomy was performed. An incision was made in the trachea and a plastic cannula tied in position. The portal vein was exposed and a plastic cannula was ligatured into position ; the 4-dimethylaminostilbene (1 ml. of the 1 per cent solution in arachis oil) was injected into the cannula. and the animal was killed 4 minutes later.

4. *Technique of section cutting*

A sample of each of three lobes of the liver was taken and frozen immediately at between −40° and −70° C. Sections were cut at 8 $\mu$ on a freezing cryostat microtome at about −20° C. Details of this procedure, which is similar to that used by Coons, Leduc and Kaplan (1951) will be discussed elsewhere.

The sections were dried over phosphorus pentoxide in an evacuated dessicator for 1 hour at −30° C. and then for 1 day at 0° C., except when used in studies on succinic dehydrogenase activity. where they were stained immediately.

5. *Histochemical methods*

(a) *Fluorescence microscopy.*—Freshly prepared frozen sections were examined by fluorescence microscopy for the presence of the fluorescent carcinogen. The apparatus was as described by Baldwin *et al.* (1960) ; the exciting light was mainly of 365 m$\mu$ wavelength.

In addition, the sections were immersed in a saturated caffeine solution to which had been added, drop by drop, a solution of about 10 mg. of 4-dimethylaminostilbene in acetone until precipitation occurred. The sections were then examined by fluorescence microscopy.

(b) *Method for demonstrating succinic dehydrogenase.*—Freshly prepared frozen sections were incubated at 37° C. in a 0·1 per cent solution of neotetrazolium in a 0·05 M phosphate buffer (Sorensen's) at pH 7·8, to which was added sodium succinate at a concentration of 0·05 M. Nitrogen was bubbled through the solution before use. The control incubation solution lacked the succinate. The method, on frozen sections, gave semi-quantitative results, depending on the time required to yield appreciable colour, or to produce the same amount of colour as a standard. After they were stained, the sections were mounted directly in Farrant's medium.

(c) *Janus Green.*—Freshly prepared frozen sections were mounted in a 0·01 per cent solution of Janus Green in 0·85 per cent sodium chloride.

(d) *Oil Red O.*—Freshly prepared frozen sections were fixed for 5 minutes in a 10 per cent solution of neutralized formalin (40 per cent Formaldehyde) containing 0·9 per cent sodium chloride. They were rinsed in distilled water, immersed in 60 per cent iso-propyl alcohol for 3 minutes and then in the Oil Red O solution (saturated Oil Red O in isopropyl alcohol 3 parts, distilled water 2 parts) for 10 minutes. They were rinsed in 60 per cent isopropyl alcohol and then in distilled water, counterstained with Harris' haematoxylin and mounted in Farrant's medium.

### RESULTS

1. *Observations by fluorescence microscopy*

(a) *Normal and control livers.*—Normal and control liver sections exhibited a faint autofluorescence ; the liver cells were very pale green with brighter green outlines and dark non-fluorescent nuclei.

(b) *Liver sections from injected animals.*—The carcinogen, dissolved in arachis oil, emitted a strong blue fluorescence under ultraviolet light—arachis oil itself was non-fluorescent.

Liver sections from animals treated with intraperitoneal injections of 4-dimethylaminostilbene showed no increase of fluorescence. In order to ensure that the carcinogen was reaching the liver, it was injected directly into the portal vein in some animals. When sections of the liver were examined, no characteristic fluorescence was seen.

(c) *" Staining " of normal and control liver sections by 4-dimethylaminostilbene.*—When normal and control liver sections were examined by fluorescence microscopy after " staining " with an aqueous suspension of 4-dimethylaminostilbene, the entire section exhibited the bright blue fluorescence of the carcinogen. This fluorescence faded uniformly and after 5 minutes the sections showed only normal autofluorescence.

(d) *" Staining " of liver sections from injected animals by 4-dimethylaminostilbene.*—Although sections of the livers of animals injected with the carcinogen did not show fluorescence due to the aminostilbene, they could be " stained " by immersion in an aqueous suspension of 4-dimethylaminostilbene ; as with the control sections, this induced fluorescence faded within 5 minutes. In addition, " stained " sections from the animal killed half an hour after injection showed bright blue fluorescent droplets in the periportal cells, which retained the fluorescence for 15 minutes, that is, for 10 minutes after the generalized fluorescence had faded. It could be demonstrated by staining with Oil Red O that these were fat droplets.

(e) *To test whether fading of the fluorescence of 4-dimethylaminostilbene was due to quenching by cellular constituents.*—Normal liver sections, fixed by formalin, and then " stained " in an aqueous suspension of 4-dimethylaminostilbene, exhibited generalized bright blue fluorescence. On repeated examination by fluorescence microscopy, this fluorescence was constant and did not fade.

Despite this result, it was apparent that the fading of the fluorescence could be due to quenching by some unknown cellular constituent which is inactivated by formalin. However, in an animal which died under anaesthetic, and which was injected intravenously into the portal vein immediately after death, the fluorescence of the carcinogen was seen strongly throughout the sinusoids and also in the cytoplasm of the liver cells in frozen sections. Hence, where it might be expected that metabolic activity, particularly oxidation, had been stopped, no fading occurred.

2. *Histochemical estimation of succinic dehydrogenase activity*

Succinic dehydrogenase activity was estimated by the rate of appearance and intensity of red stained granules in the cells. In the livers of animals $A_N$ and $A_C$, cytoplasmic granules were coloured red after half an hour's incubation, with very intense staining after 2 hours. In animal $A_{\frac{1}{2}}$ no stain was observed after half an hour and only faint coloration after 2 hours' incubation. The staining of animals $A_1$ and $A_2$ was stronger than $A_{\frac{1}{2}}$ but still weaker relative to $A_N$. The cytoplasmic granules in the liver cells of $A_6$ and $A_{24}$, however, stained as did the normal, that is the succinic dehydrogenase activity was recovered 6 hours after injection.

To assess the relative activity in $A_{\frac{1}{2}}$, sections of $A_{\frac{1}{2}}$ were incubated for 2 hours and the intensity of reaction seemed identical with that seen in sections of $A_6$ incubated for only 1 hour. Hence the succinic dehydrogenase activity in $A_{\frac{1}{2}}$ was reduced by approximately one half.

3. *Janus Green staining*

Since succinic dehydrogenase is typically a mitochondrial enzyme, it was advisable to see how far the changes in the activity of this enzyme could be correlated with morphological alteration. For this purpose, sections were stained with Janus Green. Mitochondria were seen in various forms ; filaments, rods, granules and globules could be identified, all of which stained selectively with this dye.

Filamentous mitochondria were found only in the normal and control livers. After injection of carcinogen, the mitochondria were in the form of short rods and granules. Globular forms were also seen in some of the liver cells and persisted in the livers of the animals killed 6 and 24 hours after injection. In addition green staining droplets were seen in the cytoplasm of the periportal liver cells of the animal killed half an hour after injection. These droplets stained with Oil Red O, indicating that they consisted of fat. Fat droplets were also present in the periportal cells of the liver sections from the animal killed 1 hour after injection, and to a lesser extent in the animal killed 2 hours after injection, but there were no fat droplets in the sections from the animals killed 6 and 24 hours after injection.

### DISCUSSION

1. *Fluorescence*

After intravenous or intraperitoneal injection of 4-dimethylaminostilbene, no added fluorescence can be seen in the liver cells. The blue fluorescence of 4-dimethylaminostilbene may be masked by the normal autofluorescence, or its fluorescence may be quenched, or the carcinogen may be rapidly metabolized

50

and the fluorescence destroyed. When a solution of 4-dimethylaminostilbene is viewed under ultraviolet light, the blue fluorescence is constant and does not fade, showing that it is not destroyed by ultraviolet light. Moreover, when sections are " stained " by a suspension of the carcinogen, blue fluorescence is visible and is distinct from the autofluorescence. Hence, if the fluorescence of the carcinogen were present, it would have been seen and therefore almost certainly was not masked by the autofluorescence.

Snapper *et al.* (1951) found that the fluorescence of stilbamidine was quenched by nucleic acids. That this is the explanation of the results reported in the present communication is unlikely for two reasons. Firstly, no fading occurred when a section, fixed by formalin, was " stained " by a suspension of 4-dimethylaminostilbene ; it is possible, however, that some unknown cellular constituent, responsible presumably for quenching, had been inactivated by formalin. Secondly, examination of a section, taken from an animal which had died under anaesthetic and which was injected intravenously into the portal vein immediately after death, showed brilliant fluorescence of the carcinogen which persisted and was not quenched ; in this case metabolic activity, particularly oxidation, had presumably been stopped and no fading of fluorescence was observed.

Thus it appears likely that the fading of the fluorescence that occurs after " staining " with the carcinogen, and the lack of fluorescence in the injected animals is due to rapid metabolism of the carcinogen to a non-fluorescent form. Where the " stain " is taken up preferentially by fat droplets in the liver cells, the fluorescence is destroyed less rapidly, fading after about 15 minutes, and this delay is probably due to the partial protection against metabolism afforded to the carcinogen by the fat droplets.

2. *Succinic dehydrogenase activity and Janus Green results*

After injection of 4-dimethylaminostilbene, the mitochondria lose their filamentous form and appear as rods and granules. Similarly, in the animal killed half an hour after injection, succinic dehydrogenase activity is much reduced. In the animals killed at longer intervals after injection, however, although the mitochondria still appear damaged morphologically, the enzyme activity has been restored to the normal level.

At first sight, these observations could be interpreted simply as follows : the fact that the mitochondria can be deformed grossly and yet stain well for the enzyme suggests that their morphological integrity is not essential for succinic dehydrogenase activity. Hence the reduction in the activity of this enzyme shortly after injection of the carcinogen implies a direct inhibition of the enzyme. Such an inhibition has been demonstrated by Elson (1952, 1958) for oxidized metabolites of the related carcinogen dimethylaminoazobenzene. If this suggestion is correct and if oxidized metabolites of 4-dimethylaminostilbene are present half an hour after injection, then it is not surprising that little or no fluorescence due to the intact molecule, has been observed in the liver cells.

The speed of metabolism of the 4-dimethylaminostilbene might appear excessively fast but such a rapid metabolism of the related dimethylaminoazobenzene has been demonstrated by Mueller and Miller (1948), who showed that demethylation began at 10 minutes and was almost complete at 30 minutes. It is obvious that the final proof that 4-dimethylaminostilbene is metabolized depends on biochemical investigations which are now being undertaken.

### CONCLUSIONS

After a single injection of 4-dimethylaminostilbene, neither protein binding nor permanent depression in succinic dehydrogenase activity can be demonstrated in the liver cells. It seems clear, however, that the liver cells react sharply to the carcinogen. Further injections of the carcinogen may modify this initial reaction.

### SUMMARY

1. The localization of the strongly fluorescent carcinogen 4-dimethylamino-stilbene was studied by fluorescence microscopy in frozen sections of rat liver. No characteristic fluorescence was seen after intraperitoneal or intravenous injection of the stilbene. The fluorescence faded rapidly in frozen liver sections " stained " with 4-dimethylaminostilbene, but persisted in dead or fixed liver sections. These results appeared to indicate rapid metabolism of the carcinogen and there was no evidence that the whole molecule became bound in the liver cells.

2. Morphological damage to the mitochondria of the liver cells appeared soon after injection and persisted for at least 24 hours. There was concomitant depression of succinic dehydrogenase activity soon after injection, but this recovered to normal levels in a few hours. It appeared likely that succinic dehydrogenase was directly inhibited, probably by metabolites of the carcinogen.

### REFERENCES

BALDWIN, R. W., BESWICK, J., CHAYEN, J. AND CUNNINGHAM, G. J.—(1960) *Acta Un. int. Cancr.*, **16**, 47.

COONS, A. H., LEDUC, E. H. AND KAPLAN, N. H.—(1951) *J. exp. Med.*, **93**, 173.

ELSON, L. A.—(1952) *Brit. J. Cancer*, **6**, 393.—(1958) *Brit. med. Bull.*, **14**, 161.

*Idem* AND HOCH-LIGETTI, C.—(1945) *Biochem. J.*, **40**, 380.

MILLER, E. C. AND MILLER, J. A.—(1955) *J. nat. Cancer Inst.*, **15**, 1571.

MUELLER, G. C. AND MILLER, J. A.—(1948) *J. biol. Chem.*, **176**, 535.

SNAPPER, I., SCHNEID, B., LIEBEN, F., GERBER, J. AND GREENSPAN, E.—(1951) *J. Lab. clin. Med.*, **37**, 562.